# Cardiac and systemic biological age capture complementary aging signals associated with cardiovascular disease

**DOI:** 10.3389/fcvm.2026.1877589

**Published:** 2026-08-25

**Authors:** Yiming Wang, Hao Zhu, Xiaotong Liu, Chenwen Yuan, Jing Liu, Xiaodong Wang, Yu Duan

**Affiliations:** 1 Division of Geriatric Endocrinology, the First Affiliated Hospital of Nanjing Medical University, Nanjing, China; 2 Division of Endocrinology, the First Affiliated Hospital of Nanjing Medical University, Nanjing, China

**Keywords:** biological age, blood biomarkers, cardiovascular disease, echocardiography, machine learning

## Abstract

**Background:**

Biological ages capture aging signatures associated with aging-related outcomes, but most models use systemic and unimodal indices. The added value of organ-specific and multimodal information for assessing organ-specific disease associations remains unclear.

**Methods:**

We enrolled 1,830 participants (77.81% male; age 55.87 ± 11.07 years) undergoing blood chemistry testing and echocardiography. Cardiac, systemic, and multimodal biological age models were trained using supervised machine learning with 20-fold cross-validation to predict chronological age from LASSO-selected variables among 15 echocardiographic indices, 73 blood-based indices, or their combination. Biological age acceleration (BAA) was derived using modality-specific age-bias correction functions fitted in the apparently healthy reference cohort and standardized before analysis. Associations between BAA and prevalent cardiovascular disease (CVD; ICD-10 I00–I99) were evaluated using multivariable logistic regression. Models were developed in 937 apparently healthy participants; CVD analyses compared 810 cases with 1,020 CVD-negative participants.

**Results:**

Cardiac biological age showed moderate accuracy (mean absolute error [MAE] = 6.08 years; *R*^2^ = 0.34), with A1 (late diastolic transmitral flow velocity) and septal e′ (early diastolic mitral annular velocity) as the leading SHAP contributors. Systemic biological age performed better (MAE = 4.60 years; *R*^2^ = 0.65), with estimated glomerular filtration rate and creatinine as the leading contributors. Cardiac BAA showed a numerically larger association with prevalent CVD than systemic BAA (odds ratio [OR] = 1.36, 95% confidence interval [CI] 1.23–1.52 vs. OR = 1.31, 95% CI 1.20–1.43). Cardiac and systemic biological ages showed partial overlap (*R*^2^ = 0.25). The multimodal model achieved the best age-prediction performance (MAE = 4.29 years; *R*^2^ = 0.70) and was associated with prevalent CVD (OR = 1.40, 95% CI 1.28–1.53). Subtype-specific associations were broadly directionally consistent; significance was reached for hypertension, whereas estimates for smaller subtypes remained imprecise.

**Conclusions:**

Cardiac and systemic biological age models captured complementary aging information and showed distinct strengths in chronological-age estimation and cross-sectional CVD assessment. The multimodal model achieved the lowest MAE and highest *R*^2^, supporting the complementary value of multimodal integration for biological aging assessment.

## Introduction

Population aging is a global challenge associated with the rising burden of chronic diseases, functional decline, and mortality (1, 2). Aging is a heterogeneous biological process influencing multiple dimensions (e.g., epigenetics, cellular functions, and organ structures), and individuals of the same chronological age can differ markedly in physiological function and disease susceptibility (3, 4). Instead of measuring one's aging status through passage of time from birth, biological age captures the aging signatures from blood biochemistry, omics, and imaging features, showing stronger associations with age-related outcomes (5). However, while classic biological age models built through training of massive unimodal indices (e.g., DNA methylation, blood chemistry, and imaging) showed great advances in all-cause mortality prediction, their performance in revealing organ-specific aging status and diseases is yet to be determined (6). Considering the emerging evidence regarding the asynchronous pace of aging across different organs and dimensions, the added value of organ-specific and multimodal information for evaluating organ-specific disease associations is worthy of further investigation (7).

Cardiovascular disease (CVD) is the leading cause of mortality worldwide (8). Aging is a well-established risk factor for CVD (9). Advanced age is not only associated with direct, dynamic, incremental changes in cardiac structure and function, but is also accompanied by systemic disturbances relevant to CVD (e.g., systemic inflammation (10), metabolic dysfunction (11), and hemodynamic overload (12). Although several studies have demonstrated the association between cardiac biological aging and CVD occurrence (13), few have addressed the distinct roles of primary cardiac aging and secondary cardiac aging associated with systemic aging in CVD-related phenotypes. Primary cardiac aging signatures and secondary systemic aging signals could be captured through direct cardiac examination and systemic blood tests (14, 15). Echocardiography provides a non-invasive and widely accessible assessment of cardiac changes, capturing organ-specific aging by reflecting age-related structural and functional remodeling of the heart (16). In contrast, blood-based biomarkers primarily reflect systemic metabolic, inflammatory, and hematological states and have been widely used to construct biological age models capturing whole-body aging (17).

Machine learning (ML) is transforming medical research by leveraging data-driven algorithms to model complex healthcare relationships (18). In the field of aging research, these supervised learning techniques, particularly advanced regression models, have become pivotal for the precise estimation of biological age. By accurately predicting health outcomes and biological aging, ML facilitates personalized medicine and provides critical insights into the mechanisms of aging-related diseases (19).

To clarify the relationship between biological aging and prevalent CVD, a more comprehensive understanding of the association between cardiac biological aging and CVD is of great importance. The distinct roles of primary cardiac aging-related changes and secondary alterations associated with systemic aging, as well as the complementary value of information across different modalities in cross-sectional CVD assessment, however, remain to be clarified. In this context, we developed three biological age models based on different sources of phenotypic information: a cardiac model derived exclusively from echocardiographic measures capturing cardiac structure and function, a systemic model built from blood biomarkers reflecting multi-organ physiological status, and a multimodal model integrating both echocardiographic features and blood biomarkers. The objective of this study was to (1) characterize the discrepancies between cardiac and systemic biological age models and their respective associations with CVD, and (2) evaluate the complementary value of organ-specific (cardiac) and systemic features for optimizing the multimodal biological age model.

## Methods

### Study design

**Figure 1:**
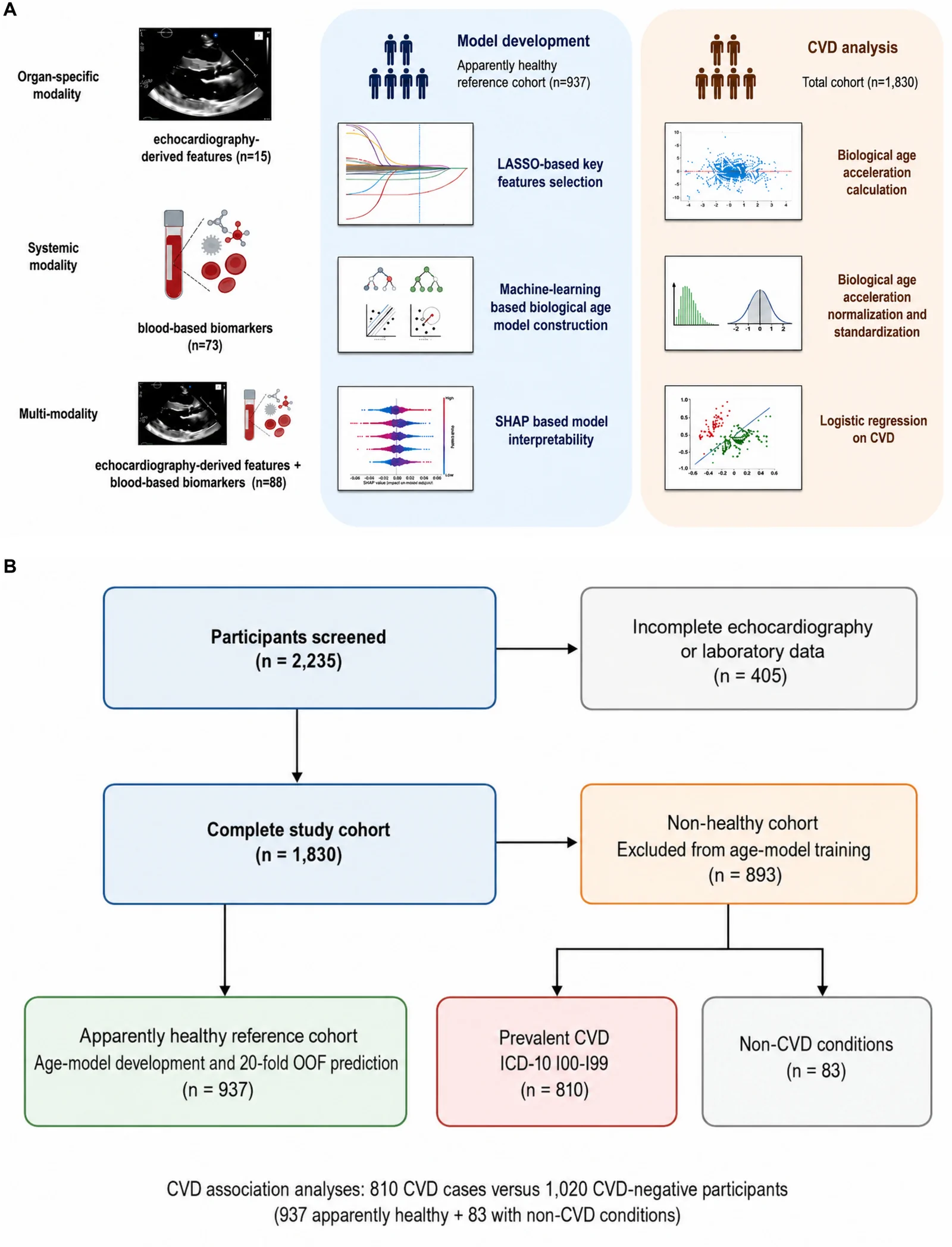
Overview of the study workflow and participant allocation. **(A)** Biological age model development and downstream analyses. **(B)** Participant flow and reconciled groups for age-model development and CVD association analyses.

This retrospective study constructed and evaluated three biological age models based on echocardiographic parameters, systemic blood biomarkers, and a combination of both. The analytical pipeline mainly consisted of two phases: (1) development and validation of the biological age models; and (2) assessment of the association between biological age acceleration and CVD. An overview of the biological age model development and downstream cardiovascular disease association analyses is shown in Figure 1A.

### Participants and data acquisition

Individuals undergoing routine health examinations at the First Affiliated Hospital of Nanjing Medical University between 2023 and 2024 were included based on the following inclusion criteria: (1) age ≥18 years; (2) availability of fully recorded routine clinical blood-based laboratory data; and (3) comprehensive echocardiographic reports. Of 2,235 individuals screened, 1,830 with complete echocardiographic and laboratory data were included. Basic information (e.g., age, sex) and blood-based biomarkers including hematological (e.g., lymphocyte count, procalcitonin), metabolic (e.g., HbA1c (Hemoglobin A1c), glucose), and biochemical (e.g., eGFR (estimated glomerular filtration rate), creatinine) indices were collected. Cardiac structure and function features (e.g., lateral e′ (early diastolic myocardial velocity derived from tissue Doppler imaging, cm/s), E/A (ratio of early to late diastolic transmitral flow velocities)) were extracted from echocardiographic examinations performed using a Philips EPIQ 7C ultrasound system and assessed by experienced echocardiography specialists. Cardiovascular disease (CVD) was defined and diagnosed based on the International Classification of Diseases, 10th Revision (ICD-10) codes spanning I00 to I99. The study was conducted in accordance with the Declaration of Helsinki and was approved by the ethics committee of the First Affiliated Hospital of Nanjing Medical University (Approval Number: 2025-SR-856).

### Data partitioning

Among the included participants, 937 individuals with neither a documented clinical diagnosis nor a recorded medication history in the institutional health-status records were used to develop the three biological age models, while non-healthy participants (*n* = 893) were excluded from model training. Supervised machine-learning models were developed within the apparently healthy reference cohort using 20-fold cross-validation to select modality-specific algorithm and hyperparameter configurations based on chronological-age estimation performance and to generate out-of-fold biological age estimates. Scaling and LASSO feature selection were refitted within the training partition of each of 20 outer folds; within each outer training partition, the LASSO regularization parameter was selected by 5-fold cross-validation. Each held-out fold was used only to generate out-of-fold biological age estimates (20). The final model was then applied to the entire cohort, including 810 participants with CVD (ICD-10 I00–I99) (21, 22) and 83 participants with non-cardiovascular conditions, enabling evaluation of biological age acceleration and its association with CVD. The CVD-negative comparison group therefore comprised 1,020 participants (Figure 1B). Baseline characteristics of the apparently healthy reference cohort and non-healthy participants are presented in Supplementary Table S1.

### Biological age model development

#### Feature selection via LASSO regression

LASSO regression was employed for feature selection, reducing dimensionality and improving interpretability by imposing an L1 penalty that shrinks uninformative coefficients to zero (23). The regularization parameter *α* was optimized using cross-validated grid search with mean absolute error (MAE) as the criterion. After refitting the model with the optimal *α*, features with non-zero coefficients were retained. Within each outer training partition, the regularization parameter *α* was selected by 5-fold cross-validation, after which the fitted scaler and selector were applied to the held-out fold.

### Machine learning models for biological age estimation

Biological age was operationalized as the age predicted by supervised regression models trained to estimate chronological age. Three biological age models were developed according to input modality: (1) 15 echocardiography-derived indices, (2) 73 blood-based laboratory indices, and (3) their multimodal combination, using multiple regression algorithms, including Random Forest, Extreme Gradient Boosting (XGBoost), Support Vector Regression (SVR), and K-Nearest Neighbors (KNN) (24). Supervised regression models were developed within the apparently healthy reference cohort using 20-fold cross-validation to select modality-specific algorithm and hyperparameter configurations based on chronological-age estimation performance and generate out-of-fold age predictions. Model performance was summarized using MAE, root mean squared error (RMSE), and coefficient of determination (*R*^2^). Within each of the 20 outer folds, scaling and LASSO selection—including 5-fold cross-validation of the LASSO regularization parameter within the outer training partition—were refitted, and the held-out fold was used only to generate out-of-fold age predictions.

### Definition and standardization of biological age acceleration

To account for systematic age-related bias in machine learning-based age prediction, biological age acceleration (BAA) was defined using a residualization approach to obtain an age-bias-corrected measure (25).

$$\mathrm{BA}A_{i}=BA_{i}\text{-}(\beta_{0}+\beta_{1}\;\times CA_{i}+\beta_{2}\;\times CA_{i}^{2})$$

Using the predicted ages, BAA was derived by removing the modality-specific relationship between predicted age and chronological age using a modality-specific linear or quadratic age-trend function. Within each of the 20 outer cross-fitting folds, linear and quadratic age-bias correction functions were compared using 10-fold cross-validation restricted to the corresponding outer training partition. A quadratic form was selected only when its quadratic term was statistically significant (two-sided *P* < 0.05) and its out-of-fold RMSE was lower than that of the linear form. Cardiac predicted age selected the quadratic form in all 20 outer folds; systemic and multimodal predicted age selected the linear form (*β*₂ = 0). The quadratic-term *P* = 0.001 was from the final cardiac fit in the full reference cohort, not a pooled foldwise *P* value. The resulting modality-specific correction was cross-fitted within the reference cohort before BAA was derived for the full cohort. Positive BAA indicates an older predicted age than expected for chronological age. Because this correction was estimated in the reference cohort, chronological age was also included in an additional sensitivity analysis of the full cohort.

For comparability across modalities, BAA was standardized prior to association analyses using a z-score transformation: the reference mean (μ_ref) and population standard deviation (σ_ref) were calculated from the cross-fitted BAA values of the 937-participant apparently healthy reference cohort.

$$\mathrm{BA}A_{z,i}=\frac{\mathrm{BA}A_{i}\text{-}\mu_{\mathrm{ref}}}{\sigma_{\mathrm{ref}}}$$

Standardized BAA was used as the primary exposure in regression analyses, allowing effect sizes to be interpreted per 1-SD increase and enabling direct comparison across modalities.

### Association analyses with CVD

For downstream analyses, the selected biological age model for each modality was applied to the full study cohort. For individuals included in model development, out-of-fold predictions were used as biological age estimates to avoid in-sample bias, whereas biological age for individuals not included in model development was generated using the trained preprocessing procedures and final model parameters. Standardized BAA was subsequently computed for all participants with available data.

The association between BAA and CVD was evaluated using multivariable logistic regression models with CVD status as the outcome. Standardized BAA was included as the primary exposure variable. Models were adjusted for prespecified covariates, including sex, smoking, drinking, height, and weight. Effect sizes were summarized as odds ratios (ORs) with 95% confidence intervals (CIs) per 1-SD increase in standardized BAA. Prespecified sensitivity analyses added chronological age, cardiometabolic covariates, and broader laboratory covariates. Medication-adjusted, sex-stratified, interaction, and CVD-subtype analyses are described below and shown in Supplementary Figures S3–S5.

### Sensitivity analyses

Medication sensitivity analyses used four binary treatment indicators: lipid-lowering, antihypertensive, glucose-lowering, and antiplatelet/anticoagulant medication. Treatment-sensitive lipid outcomes were examined using ordinary least-squares regression with HC3 heteroskedasticity-consistent standard errors, with CVD as the exposure. The covariate-adjusted model included age, sex, smoking, drinking, height, and weight; the medication-adjusted model additionally included lipid-lowering medication. The medication-adjusted BAA–CVD sensitivity model included the broad laboratory covariates and all four medication indicators; the exclusion analysis included age, sex, smoking, drinking, height, and weight and omitted lipid-lowering medication users. Sex sensitivity analyses summarized chronological-age estimation performance separately in women and men. BAA–CVD associations were estimated within each sex using the primary covariates other than sex, and BAA-by-sex interaction terms were tested in the pooled cohort. For CVD-subtype analyses, each available recorded subtype was compared with the full CVD-negative group using Firth penalized logistic regression with chronological age, sex, smoking, drinking, height, and weight as covariates. Benjamini–Hochberg false-discovery-rate adjustment was applied across subtype-by-modality tests within each analysis family, with *q* < 0.05 considered statistically significant.

### Software

All analyses were performed using Python 3.11 and standard libraries for machine learning and statistical modeling. Model interpretability was analyzed using the SHAP Python package, which provides feature attributions via Shapley Additive Explanations (SHAP) values to explain model predictions at both local and global levels (26). Statistical significance was assessed using two-sided tests with *P* < 0.05. Incremental discrimination was evaluated using 10-fold out-of-fold AUC, calibration, Brier score, decision-curve analysis, and paired bootstrap confidence intervals. The conventional clinical risk-factor base model included chronological age, sex, smoking, systolic blood pressure, diabetes, total cholesterol, and HDL-C; the secondary original covariate-adjusted base model included chronological age, sex, smoking, drinking, height, and weight; and the broad laboratory-adjusted sensitivity model included chronological age, sex, smoking, drinking, height, weight, systolic and diastolic blood pressure, diabetes, total cholesterol, triglycerides, HDL-C, LDL-C, eGFR, and HbA1c.

## Results

### Participant characteristics

Baseline characteristics of the study participants stratified by CVD status are summarized in Table 1. Compared with participants without CVD, those with CVD were generally older, had higher body weight and blood pressure levels, and showed significant differences in sex distribution, with a higher proportion of males. Lifestyle-related factors also differed between groups, with higher prevalence of smoking and alcohol consumption among participants with CVD, and cardiometabolic comorbidities, including hypertension and diabetes mellitus, were more common in the CVD group. These characteristics describe the study population and provide context for subsequent analyses. The comparison included 810 CVD cases and 1,020 CVD-negative participants, including the 83 participants with non-cardiovascular conditions.

**Table 1:** Baseline characteristics according to CVD status.

Variables	Total (*n* = 1,830)	Without CVD (*n* = 1,020)	With CVD (*n* = 810)	*P*
Age	55.87 ± 11.07	52.63 ± 10.09	59.95 ± 10.90	<0.001
Height (cm)	167.95 ± 8.21	167.49 ± 9.14	168.53 ± 6.82	0.005
Weight (kg)	71.61 ± 11.57	69.61 ± 11.17	74.14 ± 11.58	<0.001
SBP (mmHg)	126.14 ± 15.83	121.90 ± 15.00	131.47 ± 15.23	<0.001
DBP (mmHg)	82.19 ± 10.22	80.23 ± 9.81	84.65 ± 10.19	<0.001
Sex, *n* (%)				<0.001
Female	406 (22.19)	303 (29.71)	103 (12.72)	
Male	1,424 (77.81)	717 (70.29)	707 (87.28)	
Smoking, *n* (%)				0.003
No	1309 (71.53)	758 (74.31)	551 (68.02)	
Yes	521 (28.47)	262 (25.69)	259 (31.98)	
Drinking, *n* (%)				0.043
No	805 (43.99)	470 (46.08)	335 (41.36)	
Yes	1025 (56.01)	550 (53.92)	475 (58.64)	
Hypertension, *n* (%)				Not applicable
No	1174 (64.15)	1020 (100.00)	154 (19.01)	
Yes	656 (35.85)	0 (0.00)	656 (80.99)	
Diabetes, *n* (%)				<0.001
No	1615 (88.25)	952 (93.33)	663 (81.85)	
Yes	215 (11.75)	68 (6.67)	147 (18.15)	

CVD, cardiovascular disease; SBP, systolic blood pressure; DBP, diastolic blood pressure. Continuous variables are presented as mean ± standard deviation (SD), and categorical variables are presented as *n* (%). *P* values were calculated using Welch's *t*-test for continuous variables and the Pearson chi-square test for categorical variables. Since hypertension was included in the composite CVD definition based on ICD-10 codes I00–I99, its status was not independent of the group definition. Therefore, no between-group statistical test was performed, and the corresponding *P* value is not applicable.

### Key features in echocardiographic, blood, and multimodal data

A total of 15 echocardiography-derived features and 73 blood-based indices were considered as candidate predictors, with representative variables shown in Table 2. These variables were subsequently subjected to foldwise LASSO regression within the 20-fold outer cross-validation framework against chronological age in the apparently healthy reference cohort to identify age-related predictors (Supplementary Figure S1). When the candidate space was restricted to echocardiographic indices, feature selection consistently concentrated on a compact set of measures dominated by left ventricular diastolic function. The retained variables primarily reflected transmitral inflow and tissue Doppler relaxation, including A1 (late diastolic transmitral flow velocity), septal e′ (early diastolic mitral annular velocity at the septal side), lateral e′, and composite diastolic indices such as E/A and E/e′ (ratio of early transmitral flow velocity to early diastolic myocardial velocity), suggesting that diastolic function carried the strongest age-informative signal within the echocardiographic domain.

**Table 2:** Representative variables according to reference-cohort status.

Cardiac variables	Total (*n* = 1,830)	Apparently healthy (*n* = 937)	Non-healthy (*n* = 893)	*P*
A1, cm/s	72.19 ± 19.69	67.83 ± 18.37	76.77 ± 19.99	<0.001
Septal e’, cm/s	7.57 ± 2.11	8.27 ± 2.20	6.82 ± 1.71	<0.001
Lateral e’, cm/s	10.30 ± 2.90	11.27 ± 2.95	9.28 ± 2.47	<0.001
E/e'	8.08 ± 2.19	7.57 ± 1.87	8.61 ± 2.38	<0.001
E/A	1.03 ± 0.88	1.10 ± 0.36	0.96 ± 1.20	<0.001
LVDd, mm	45.81 ± 4.25	45.57 ± 4.14	46.06 ± 4.35	0.014
LVDs, mm	29.20 ± 2.85	29.06 ± 2.68	29.34 ± 3.00	0.038

Systemic variables	Total (*n* = 1,830)	Apparently healthy (*n* = 937)	Non-healthy (*n* = 893)	*P*
Hb, g/L	147.37 ± 13.60	146.00 ± 14.03	148.81 ± 12.99	<0.001
PLT, ×10⁹/L	217.38 ± 52.85	222.39 ± 50.66	212.12 ± 54.59	<0.001
ALT, U/L	25.25 ± 22.01	23.24 ± 18.93	27.36 ± 24.68	<0.001
AST, U/L	25.11 ± 11.88	23.96 ± 9.60	26.33 ± 13.78	<0.001
TP, g/L	72.17 ± 4.14	72.26 ± 4.06	72.08 ± 4.22	0.374
eGFR, mL/min/1.73 m^2^	91.78 ± 16.59	96.21 ± 14.41	87.14 ± 17.44	<0.001
TC, mmol/L	5.09 ± 1.06	5.35 ± 0.96	4.82 ± 1.09	<0.001
TG, mmol/L	1.74 ± 1.46	1.63 ± 1.44	1.86 ± 1.47	<0.001
HDL-C, mmol/L	1.34 ± 0.30	1.40 ± 0.31	1.28 ± 0.28	<0.001
LDL-C, mmol/L	3.18 ± 0.77	3.37 ± 0.69	2.98 ± 0.79	<0.001
CK, U/L	122.19 ± 222.60	114.70 ± 83.75	130.04 ± 306.80	0.149
LDH, U/L	203.08 ± 35.82	202.28 ± 35.24	203.93 ± 36.42	0.324
Ca, mmol/L	2.34 ± 0.09	2.33 ± 0.09	2.34 ± 0.09	0.001
K, mmol/L	4.06 ± 0.28	4.08 ± 0.27	4.04 ± 0.30	0.002
FT3, pmol/L	5.04 ± 0.81	5.03 ± 0.72	5.05 ± 0.90	0.696
FT4, pmol/L	17.45 ± 2.49	17.41 ± 2.47	17.48 ± 2.50	0.551
CEA, μg/L	2.04 ± 1.38	1.87 ± 1.24	2.22 ± 1.50	<0.001

CVD, cardiovascular disease; A1, late diastolic transmitral flow velocity; septal e′, early diastolic mitral annular velocity at the septal side; lateral e′, early diastolic mitral annular velocity at the lateral side; E/e′, ratio of early diastolic transmitral flow velocity to early diastolic mitral annular velocity; E/A, ratio of early to late diastolic transmitral flow velocity; LVDd, left ventricular end-diastolic diameter; LVDs, left ventricular end-systolic diameter; Hb, hemoglobin; PLT, platelet count; ALT, alanine aminotransferase; AST, aspartate aminotransferase; TP, total protein; eGFR, estimated glomerular filtration rate; TC, total cholesterol; TG, triglycerides; HDL-C, high-density lipoprotein cholesterol; LDL-C, low-density lipoprotein cholesterol; CK, creatine kinase; LDH, lactate dehydrogenase; Ca, calcium; K, potassium; FT3, free triiodothyronine; FT4, free thyroxine; CEA, carcinoembryonic antigen. Continuous variables are presented as mean ± standard deviation (SD). *P*-values compare the apparently healthy reference cohort with the non-healthy cohort using Welch's *t*-test.

In contrast, applying the same framework to blood biomarkers yielded a broader systemic profile spanning multiple physiological domains. The age-related signal was most prominently characterized by renal function markers (filtration and creatinine indices, including urine ratio-based measures), accompanied by hepatic function measures reflecting synthetic capacity and hepatocellular injury, and metabolic and electrolyte markers capturing glycemic status and key ions. Additional contributions arose from inflammatory or immune cell indicators and a subset of tumor-associated markers, collectively indicating that chronological aging in blood was reflected by coordinated variation across multi-organ physiology rather than a single pathway.

When echocardiographic and blood variables were modeled jointly, the retained multimodal predictors reflected complementary information from both modalities. Systemic markers from renal, hepatic, and metabolic domains remained central, while key diastolic echocardiographic features (A1, septal and lateral e′, and E/A) were preserved, indicating that organ-specific functional aging signals from echocardiography provided additional information beyond systemic physiology captured by blood biomarkers.

### Performance of cardiac, systemic and multimodal models

**Figure 2:**
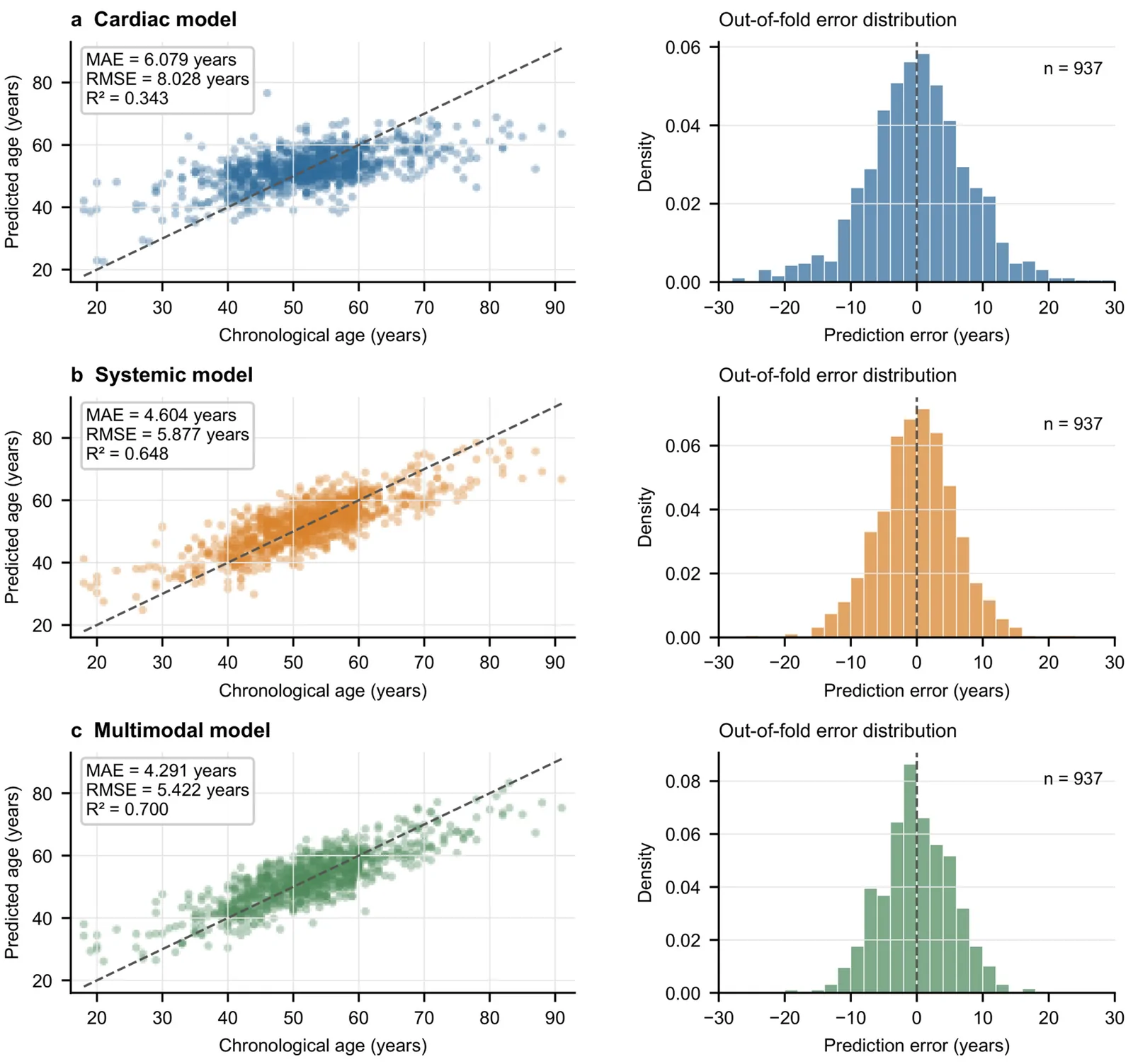
Out-of-fold chronological-age estimation performance for the selected cardiac, systemic, and multimodal models. Left panels show predicted versus chronological age; right panels show histograms of prediction errors. **(a)** Cardiac; **(b)** systemic; **(c)** multimodal.

Model performance was assessed using out-of-fold (OOF) predictions from cross-validation, with chronological age as the prediction target (Figure 2; Table 3). For the cardiac model, the K-nearest neighbors regressor achieved the best performance among the evaluated algorithms, with an RMSE of 8.028 years, an MAE of 6.079 years, and *R*^2^ = 0.343 (Figure 2a), indicating a moderate ability of echocardiographic features to capture age-related variation. For the systemic model, XGBoost provided greater predictive accuracy, with an RMSE of 5.877 years, an MAE of 4.604 years, and *R*^2^ = 0.648 (Figure 2b), supporting the contribution of blood biomarkers to the characterization of systemic aging. Integration of echocardiographic and blood-based features further improved age estimation, with the multimodal XGBoost model achieving the best overall performance (RMSE = 5.422 years, MAE = 4.291 years, and *R*^2^ = 0.700; Figure 2c). OOF prediction errors were centered near zero across all three modalities, suggesting limited systematic bias in age estimation.

**Table 3:** Out-of-fold prediction performance of three biological age models.

Modality/selected regressor	RMSE	MAE	*R* ^2^
Cardiac — K-Nearest Neighbors	8.028	6.079	0.343
Systemic — XGBoost	5.877	4.604	0.648
Multimodal — XGBoost	5.422	4.291	0.700

Performance was evaluated using out-of-fold predictions from cross-validation in the apparently healthy reference cohort. Lower RMSE and MAE and higher *R*^2^ indicate better chronological-age estimation. RMSE, root mean squared error; MAE, mean absolute error; *R*^2^, coefficient of determination; XGBoost, extreme gradient boosting; K-Nearest Neighbors, KNN.

### SHAP analysis in three prediction models

**Figure 3:**
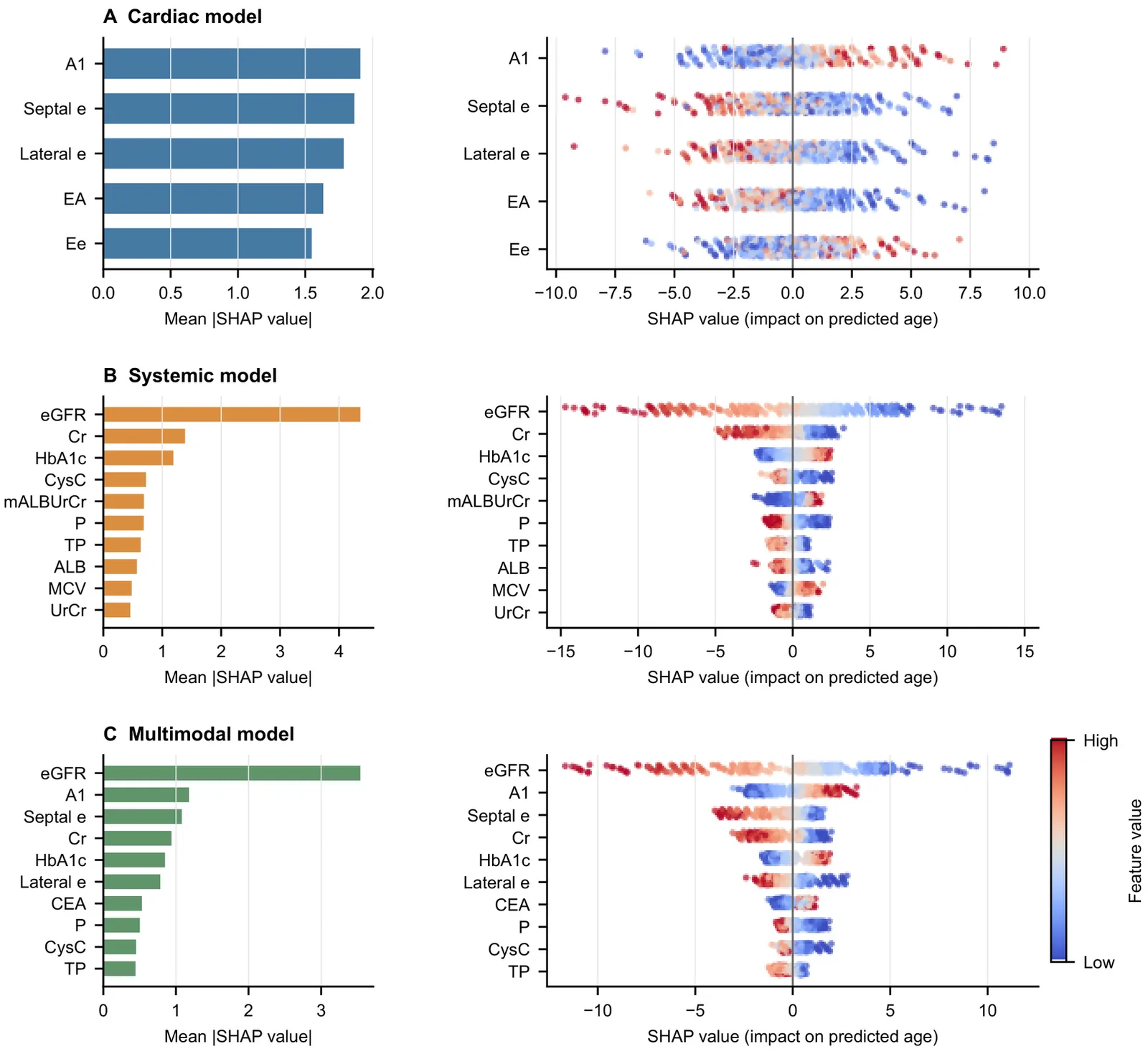
SHAP feature importance and directionality for cardiac, systemic, and multimodal biological age models. **(A)** Cardiac; **(B)** systemic; **(C)** multimodal. A1, peak late diastolic transmitral flow velocity; ALB, albumin; CEA, carcinoembryonic antigen; Cr, creatinine; CysC, cystatin C; eGFR, estimated glomerular filtration rate; HbA1c, hemoglobin A1c; MCV, mean corpuscular volume; P, serum phosphorus; SHAP, Shapley additive explanations; TP, total protein; UrCr, urinary creatinine.

Model interpretability was assessed using SHAP to quantify the contribution of individual features to predicted biological age within each modality (Figure 3). In the cardiac model, the largest SHAP contributions were consistently attributed to diastolic-function-related indices. A1, septal and lateral e′, E/A, and E/e′ emerged as the highest-ranked model attributions and contributed most strongly to the model's age estimates. In the systemic model, SHAP importance was dominated by systemic physiological markers, led by renal function indicators such as eGFR together with creatinine-related measures, followed by glycometabolic markers including HbA1c and glucose, with additional contributions from liver function measures such as albumin and aminotransferase-related indices.

In the multimodal model, the highest SHAP contributions arose from blood biomarkers, with eGFR ranking first; other renal-function measures (creatinine-related indices) and glycometabolic markers (e.g., HbA1c) also remained consistently important; alongside these, several echocardiographic diastolic indices, including A1, septal e′, lateral e′, and E/A, also occupied top-ranked positions, showing that the model used information from systemic signals and organ-specific cardiac functional features, with both modalities contributing key information to the prediction. SHAP values were interpreted as predictive attributions rather than causal effects.

### Association of model-predicted BAA with the presence of CVD

**Figure 4:**
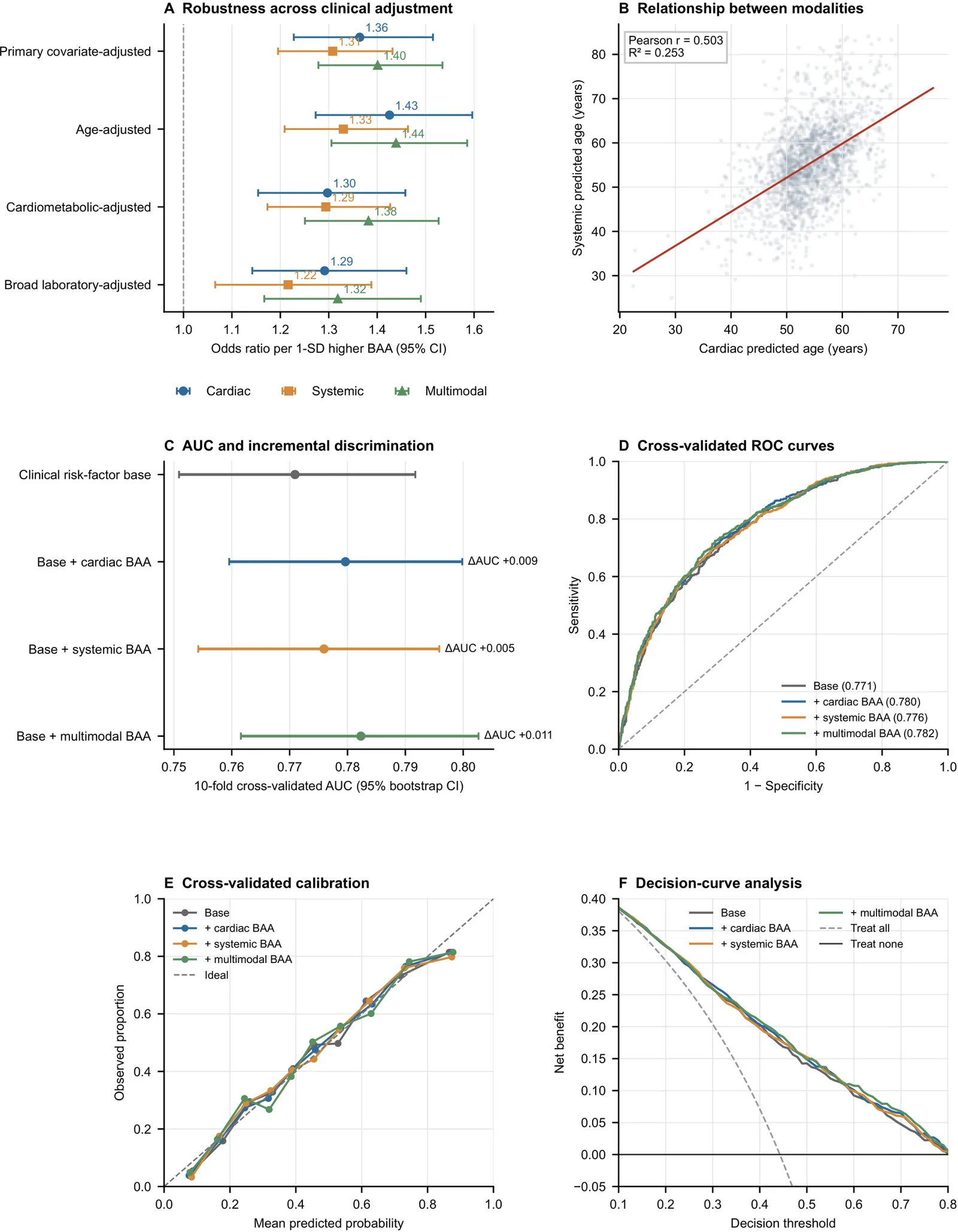
**(A)** forest plot of ORs (95% CIs) for associations between BAA and prevalent CVD across progressive clinical adjustment. The primary covariate-adjusted model included sex, smoking, drinking, height, and weight; the age-adjusted model additionally included chronological age; the cardiometabolic-adjusted model additionally included systolic and diastolic blood pressure and diabetes; and the broad laboratory-adjusted sensitivity model further included total cholesterol, triglycerides, HDL-C, LDL-C, eGFR, and HbA1c. **(B)** Relationship between cardiac and systemic biological ages (Pearson *r* = 0.50; *R*^2^ = 0.25). **(C)** Ten-fold cross-validated AUC estimates, 95% bootstrap CIs, and AUC increments after adding each BAA measure to the conventional clinical risk-factor base model. **(D)** ROC curves based on the same out-of-fold probabilities. **(E)** Cross-validated calibration of the base model and BAA extensions. **(F)** Decision-curve analysis across the displayed threshold-probability range. The conventional clinical risk-factor base model included chronological age, sex, smoking, systolic blood pressure, diabetes, total cholesterol, and HDL-C. BAA, biological age acceleration; CI, confidence interval; AUC, area under the receiver operating characteristic curve; ROC, receiver operating characteristic.

Biological age acceleration was derived for each modality using the selected age-bias correction function and was then standardized (z-score) for downstream analyses. In the corresponding plots of BAA versus chronological age based on residualized out-of-fold predictions, BAA values were symmetrically distributed around zero and showed no clear linear trend across the age range, supporting that the residualization procedure effectively reduced inherent age-related bias in the raw predicted biological age estimates (Supplementary Figure S2). In multivariable logistic regression adjusting for sex, smoking, drinking, height, and weight, higher BAA was consistently associated with greater odds of prevalent CVD across all three models. Cardiac BAA showed a significant positive association with CVD (OR per 1-SD increase = 1.36, 95% CI 1.23–1.52, *P* < 0.001) (Figure 4A), and systemic BAA was similarly associated (OR = 1.31, 95% CI 1.20–1.43, *P* < 0.001). Multimodal BAA was also associated with prevalent CVD (OR = 1.40, 95% CI 1.28–1.53, *P* < 0.001). Together, these results indicate that accelerated biological aging captured from echocardiography, blood biomarkers, and their integration is linked to the presence of CVD, supporting the relevance of both organ-specific and systemic aging signals in CVD-related phenotypes. The multimodal model combined the two sources while retaining a CVD association comparable to the cardiac model and stronger chronological-age performance than either unimodal model. Furthermore, cardiac and systemic predicted ages were moderately correlated (Pearson *r* = 0.50; *R*^2^ = 0.25; Figure 4B), indicating partial statistical overlap without establishing biological independence. Echocardiographic and blood biomarkers provided complementary information for cross-sectional CVD discrimination. Adding multimodal BAA increased the conventional clinical risk-factor model AUC from 0.771 to 0.782 (ΔAUC 0.011, 95% CI 0.004–0.019); associations were strongest for hypertension, whereas other subtype analyses were exploratory (Supplementary Figure S5). Results remained robust across sensitivity analyses. For cardiac, systemic, and multimodal BAA, respectively, ORs per 1-SD increase were 1.43 (95% CI 1.27–1.60), 1.33 (1.21–1.46), and 1.44 (1.31–1.59) after age adjustment; 1.30 (1.15–1.46), 1.29 (1.17–1.43), and 1.38 (1.25–1.53) after cardiometabolic adjustment; and 1.29 (1.14–1.46), 1.22 (1.07–1.39), and 1.32 (1.17–1.49) after broad laboratory adjustment (Figure 4A).

Among participants with CVD, the mean LDL-C concentration was 2.57 mmol/L in lipid-lowering medication users (*n* = 311) and 3.20 mmol/L in nonusers (*n* = 499). The corresponding mean total cholesterol concentrations were 4.28 and 5.11 mmol/L, respectively. In ordinary least-squares models using HC3 heteroskedasticity-consistent standard errors, additional adjustment for lipid-lowering medication use attenuated the CVD coefficient for LDL-C from −0.35 mmol/L (95% CI, −0.43 to −0.28; *P* < 0.001) to −0.15 mmol/L (95% CI, −0.23 to −0.07; *P* < 0.001). The corresponding coefficient for total cholesterol was attenuated from −0.43 mmol/L (95% CI, −0.53 to −0.32; *P* < 0.001) to −0.16 mmol/L (95% CI, −0.27 to −0.05; *P* = 0.004). In the medication-adjusted sensitivity models, the OR for prevalent CVD per 1-SD increase in BAA was 1.27 (95% CI, 1.06–1.51; *P* = 0.008) for cardiac BAA, 1.29 (95% CI, 1.06–1.57; *P* = 0.011) for systemic BAA, and 1.25 (95% CI, 1.05–1.49; *P* = 0.012) for multimodal BAA (Supplementary Figure S3).

Sex-stratified multimodal age-prediction performance was similar in women and men (MAE, 4.19 vs. 4.33 years; RMSE, 5.36 vs. 5.45 years; *R*^2^, 0.70 vs. 0.70). Corresponding BAA–CVD ORs in women versus men were 1.29 (95% CI 0.99–1.67) versus 1.38 (1.23–1.55) for cardiac BAA, 1.35 (1.08–1.68) versus 1.30 (1.18–1.43) for systemic BAA, and 1.29 (1.02–1.63) versus 1.42 (1.28–1.57) for multimodal BAA. No sex interaction was detected ($P_{interaction}$ = 0.807, 0.652, and 0.628, respectively; Supplementary Figure S4), although estimates in women were less precise because of the smaller sample.

In subtype analyses, hypertension accounted for 656 of 810 CVD cases; the ORs were 1.55 (95% CI 1.37–1.75) for cardiac, 1.38 (95% CI 1.24–1.52) for systemic, and 1.54 (95% CI 1.39–1.71) for multimodal BAA (Supplementary Figure S5). Coronary heart disease (52 cases), structural heart disease (53 cases), and arrhythmia (32 cases) were analyzed separately. Only the hypertension findings retained statistical support after multiplicity adjustment. Estimates for the smaller subtypes were broadly directionally consistent with the main finding.

For the conventional clinical risk-factor model, the base AUC was 0.771 (95% CI, 0.751–0.792), increasing to 0.780, 0.776, and 0.782 after adding cardiac, systemic, and multimodal BAA, respectively (Figures 4C, D). In the conventional clinical risk-factor model, adding multimodal BAA modestly reduced the Brier score from 0.194 to 0.189, while the calibration slope remained essentially unchanged (0.973 vs. 0.972; Figures 4E, F). For multimodal BAA, the AUC increased from 0.740 to 0.758 in the original covariate-adjusted base model and from 0.791 to 0.796 in the broad laboratory-adjusted sensitivity model (Supplementary Figure S6). Overall, BAA provided modest incremental discrimination, with the greatest improvement observed for multimodal BAA.

## Discussion

Biological aging is associated with many aging-related diseases (27) and has been identified as a heterogeneous process across various dimensions and organs (28), yet major measurements of biological age focused on limited data modalities, oversimplifying the aging process (29). In this study, we employed machine learning methods and constructed biological age models from echocardiography-derived features, blood-based biomarkers, and their combination to characterize biological aging from both organ-specific and systemic perspectives and to estimate their associations with CVD. Specifically, we (1) characterized key indices associated with age across different modalities; (2) observed distinct and complementary patterns of systemic and organ-specific biological information derived from different modalities in estimating chronological age; (3) demonstrated that multimodal BAA was associated with prevalent CVD and that the multimodal model improved chronological-age estimation.

Prior studies have developed a variety of diagnostic approaches for CVD based on blood biomarkers and cardiac structural alterations (e.g., proBNP, EF, C-reactive protein) (30). However, recent studies have indicated that for age-related cardiovascular diseases, when the diagnosis is established using conventional blood biomarkers or cardiac structural indices, irreversible structural alterations of the cardiovascular system have often already developed (31), providing context for evaluating complementary markers of cross-sectional CVD-related phenotypes. Chronological age is a well-established risk factor for CVD, including heart failure, atherosclerosis, and hypertension (9). The aging cardiovascular system undergoes a series of profound pathological alterations across multiple dimensions (e.g., cellular and molecular senescence, chronic low-grade inflammation and calcium handling dysfunction at the cellular and molecular level (32); concentric left ventricular hypertrophy, diffuse myocardial fibrosis and valvular calcification at the structural level; left ventricular diastolic dysfunction, reduced contractile reserve and vascular compliance decline at the functional level). Capturing these signals enables researchers to evaluate the biological age of the cardiovascular system which may complement chronological age in cross-sectional CVD assessment (33). In our study, by constructing biological age models, we also observed an association between biological age and CVD. Furthermore, through LASSO regression on chronological age, we identified key age-related signatures from echocardiographic features and blood-based indices. Among echocardiographic features, we determined that diastolic indices such as E/A, E/e′, septal and lateral e′, and A1 velocity were the highest-ranked age-related features, consistent with age-associated myocardial stiffening, impaired relaxation, and increased dependence on atrial contraction for ventricular filling, suggesting that diastolic function carried substantial age-related information in the cardiac model. Notably, blood-based biomarkers including proBNP are not sensitive to early and subclinical diastolic dysfunction. In comparison, among blood-based biomarkers, indices reflecting the functional status of multiple organs were identified as age-related correlates of the aging process. This multi-organ pattern is consistent with the systemic nature of aging, being associated with the onset and progression of multiple age-related comorbidities (34). In addition, the set of indices screened by single-modality LASSO regression (blood-only or echocardiography-only) differed from that identified by the multimodal LASSO model integrating both data types. This discrepancy was an expected finding driven by the altered variable space, cross-modality collinearity, and differential regularization effects between models, and further reflected the complementary nature of systemic and organ-specific aging biomarkers (6).

Although biological age estimation has enabled researchers to make substantial advances in the assessment of aging-related diseases, the increasing emergence of biological age models developed from distinct data modalities has highlighted two critical unresolved questions. (1) While these models—even those built on vastly disparate data types (e.g., DNA methylation age derived from tissue-based methylation profiling, and phenotypic age based on multi-organ functional indicators)—consistently achieve robust associations with aging-related outcomes (35), it remains unclear whether they capture the same underlying biological construct of aging. (2) A growing body of evidence has demonstrated that organs age at heterogeneous paces, with reciprocal interactions between the aging trajectories of individual organs (36). This raises fundamental questions: do organ-specific biomarkers hold unique, non-redundant biological value, and can they be replaced by multi-organ indicators from other bodily systems? In our study, we constructed and compared the cardiac biological age model, the systemic biological age model, and the multimodal biological age model using LASSO-selected key features. We found that the 5-variable cardiac model had inferior chronological-age estimation performance (lower *R*^2^, higher MAE) than the systemic and multimodal models (53 and 56 retained variables, respectively). Notably, this performance gap may reflect broader biological information coverage in the systemic and multimodal models, although the models differed in the number of retained variables. While the cardiac biological age model captured cardiac-specific aging signatures (37), the systemic biological age model reflected multi-system, whole-body aging trajectories, enabling more comprehensive age prediction (38). In addition, the multimodal biological age model achieved the best performance with a similar variable count (all five cardiac features remained) to the systemic biological age model, suggesting the complementary value of systemic and organ-specific aging biomarkers (6).

Biological age acceleration has been studied as a marker associated with mortality and morbidity (39). While all-cause mortality has been conventionally adopted as the primary outcome endpoint in prior studies, emerging evidence from recent investigations has demonstrated a robust correlation between organ-specific aging and the development of organ-specific diseases (38). However, there remains a critical paucity of research that quantitatively compares the differential associations of global BAA versus organ-specific BAA with organ-specific diseases. In our study, we calculated and normalized BAA from the cardiac, the systemic, and the multimodal biological age models to evaluate their associations with CVD. Cardiac, systemic, and multimodal BAA were each associated with prevalent CVD; effect sizes for cardiac and multimodal BAA were similar and larger than that for systemic BAA in the primary covariate-adjusted model. Because the cardiac model was derived from echocardiographic measures, cardiac BAA reflects age-related variation in the measured cardiac structure and function, particularly diastolic indices; it should not be interpreted as directly measuring unobserved processes such as myocardial fibrosis (40). In contrast, systemic BAA predominantly reflects aging-related variation across multiple organs and biological pathways and may not fully represent cardiac-specific target-organ changes (41). Meanwhile, the multimodal BAA combines two information domains of aging-related CVD: the systemic aging milieu associated with cardiovascular damage, and the cardiac-specific structural and functional degeneration that reflects cardiac structural and functional changes (42). Taken together, these findings support the interpretation that systemic and cardiac-specific aging biomarkers provide distinct and complementary biological information (43).

Several limitations of this study should be noted. First, the cross-sectional design of our study precludes the establishment of causal temporality between BAA and prevalent CVD, and we were unable to validate the predictive value of our models for future incident CVD events. Second, our sample size of 1,830 participants, while sufficient for the primary analyses, had limited power for detailed subgroup analyses, and the single-center study design may restrict the generalizability of our findings to other populations. Third, the apparently healthy reference cohort was defined by the absence of documented clinical diagnoses and recorded medication use in the available institutional records, rather than by comprehensive screening for occult disease. Accordingly, undiagnosed or subclinical conditions could not be excluded, and some participants with unrecognized conditions may have been included in the reference cohort. Finally, our models were only constructed using blood biomarkers and echocardiographic indices, without incorporating other aging-related data modalities, which may limit the comprehensive characterization of whole-body aging. Despite these limitations, our study still provides important evidence for the complementary value of systemic and cardiac-specific aging biomarkers, and supports further evaluation of multimodal biological age in CVD assessment (43). The models have not been externally validated. Hypertension comprised most CVD cases, and the heterogeneous ICD-10 I00–I99 composite limits subtype-specific interpretation. The male-predominant sample reduced the precision of female-specific estimates. Pretreatment measurements, medication dose, treatment duration, and adherence were unavailable, so residual treatment-related confounding remains possible.

In conclusion, we developed and compared echocardiography-derived cardiac-specific, blood-based systemic, and multimodal biological age models. We observed distinct and complementary patterns of systemic and organ-specific aging biomarkers, with the multimodal model achieving optimal chronological age prediction performance (44). Cardiac, systemic, and multimodal biological age acceleration were each associated with prevalent CVD, while the multimodal model achieved the best chronological-age estimation performance. Our findings support the utility of multimodal integrated models for aging characterization and cross-sectional CVD assessment. These cross-sectional associations primarily reflected the hypertension component of the composite CVD outcome and require validation in other cardiovascular subtypes.

## Data Availability

The original contributions presented in the study are included in the article/Supplementary Material, further inquiries can be directed to the corresponding authors.

## Supplementary material

The Supplementary Material for this article can be found online at: https://www.frontiersin.org/articles/10.3389/fcvm.2026. 1877589/full#supplementary-material
