# Supplementary material for: Cardiac and systemic biological age capture complementary aging signals associated with cardiovascular disease
Source: Front Cardiovasc Med. 2026 Aug 25;13:1877589. doi: 10.3389/fcvm.2026.1877589 (PMC13550194; doi:10.3389/fcvm.2026.1877589)
Supplement: Supplementary file 1 [file Supplementaryfile1.docx]

**Supplementary Figures**

Fig. S1. LASSO tuning curves and coefficient paths for the cardiac, systemic, and multimodal biological age models. (A) Cardiac; (B) systemic; (C) multimodal.

Fig. S2. Biological age acceleration plotted against chronological age after modality-specific age-bias correction in the apparently healthy reference cohort.

Fig. S3. Medication sensitivity analyses for the associations of systemic and multimodal BAA with prevalent CVD among 810 cases and 1,020 CVD-negative participants. Odds ratios and 95% confidence intervals are shown for the age-adjusted model, the broad laboratory-adjusted sensitivity model, the medication-adjusted sensitivity model, and the sensitivity analysis excluding lipid-lowering medication users. Odds ratios are presented per 1-SD higher BAA. The age-adjusted model included sex, smoking, drinking, height, weight, and chronological age; later models added the stated laboratory covariates and four medication indicators.

Fig. S4. Sex-stratified chronological-age estimation performance and BAA-CVD associations. Panel A displays MAE bars with R² annotations in women and men; RMSE values were 6.91 and 7.63 years for cardiac, 6.00 and 5.75 years for systemic, and 5.36 and 5.45 years for multimodal models, respectively. Panel B shows odds ratios with 95% confidence intervals per 1-SD higher BAA from sex-stratified logistic regression adjusted for smoking, drinking, height, and weight. BAA-by-sex interaction P values were obtained by adding the corresponding product term to the full-cohort model. Women comprised 406 of 1,830 participants and 103 of 810 CVD cases; the smaller female sample therefore yielded wider confidence intervals. BAA, biological age acceleration; CI, confidence interval; MAE, mean absolute error; RMSE, root mean squared error.

Fig. S5. Subtype-specific associations using 1,020 CVD-negative participants as controls. The hypertension subtype included 656 cases and showed statistically significant associations across all three BAA measures. Estimates for the smaller subtypes were broadly directionally consistent with the main finding: all multimodal and most cardiac and systemic point estimates were above 1, although the analyses were exploratory and the estimates imprecise. Models were fitted using Firth penalized logistic regression and adjusted for age, sex, smoking, drinking, height, and weight. Odds ratios are shown per 1-SD higher BAA with 95% confidence intervals. Benjamini-Hochberg false-discovery-rate correction was applied across the three modalities within each subtype family.

Fig. S6. Incremental discrimination across the original covariate-adjusted, conventional clinical risk-factor base, and broad laboratory-adjusted sensitivity models. Points show changes in 10-fold out-of-fold AUC and horizontal lines show paired bootstrap 95% confidence intervals from 2,000 resamples. All scenarios used the same 810 CVD cases and 1,020 CVD-negative participants; covariates are defined in Methods, and BAA effects are evaluated per 1-SD increase.
